# Common Criteria Related Security Design Patterns for Intelligent Sensors—Knowledge Engineering-Based Implementation

**DOI:** 10.3390/s110808085

**Published:** 2011-08-17

**Authors:** Andrzej Bialas

**Affiliations:** Institute of Innovative Technologies EMAG, 40-189 Katowice, Leopolda 31, Poland; E-Mail: a.bialas@emag.pl; Tel.: +48-32-2007-700; Fax: +48-32-2007-701

**Keywords:** Common Criteria, information security, IT security development, intelligent sensor, design pattern, knowledge engineering

## Abstract

Intelligent sensors experience security problems very similar to those inherent to other kinds of IT products or systems. The assurance for these products or systems creation methodologies, like Common Criteria (ISO/IEC 15408) can be used to improve the robustness of the sensor systems in high risk environments. The paper presents the background and results of the previous research on patterns-based security specifications and introduces a new ontological approach. The elaborated ontology and knowledge base were validated on the IT security development process dealing with the sensor example. The contribution of the paper concerns the application of the knowledge engineering methodology to the previously developed Common Criteria compliant and pattern-based method for intelligent sensor security development. The issue presented in the paper has a broader significance in terms that it can solve information security problems in many application domains.

## 1. Introduction

Progress in sensor technology and increased interoperability have made sensor systems more complex, more efficient and allowed them to reach new application domains. Sensor systems are integrated with other complex IT systems, creating huge collaborative systems. The flow of the collected information, its processing and decisions based on these data, co-ordination and co-operation with complex IT systems–all are based on dependable solutions. Information security and operation continuity issues are very important for sensors and sensor systems, especially for those working in high-risk environments. Numerous collaborative sensors applications operate in these environments, so their security issues should be considered with special attention during their development.

The paper concerns generally the information technology (IT) security development methodology compliant with ISO/IEC 15408 Common Criteria (CC) [1,2], but is focused on the implementation of this methodology in intelligent sensors. Earlier papers [3,4] introduced the patterns-based IT security development method and discussed its validation on two different kinds of sensors, *i.e*., a mote-based medical sensor remotely monitoring patients and a methane detector working in a coal mine environment. The objective of this paper is to improve the introduced method by using possibilities and advantages offered by the knowledge engineering approach.

The risk inherent in IT products or systems, including sensor systems, can be mitigated by different measures, such as the measures built into these products and systems. The question is whether the users can rely on these measures in critical circumstances, when certain threats occur. In this case the methodology providing assurance, like the Common Criteria methodology can be helpful. Assurance is understood as a situation when an IT product or system meets its security objectives which are related to the built-in measures or the measures applied in the product or system operational environment. The term assurance means that the implemented measures will be able to counter threats when they occur. According to the Common Criteria paradigm the source of assurance are: the rigour applied during the development and manufacturing processes, independent third-party evaluation as well as the operation and maintenance according to the received certificate. These specially developed and evaluated IT products and systems can operate in a higher risk environment. The Common Criteria standard, broadly used for years and still being improved, can be applied to different groups of IT products (hardware, software, firmware) and systems. Each of them is called TOE, *i.e*., target of evaluation. Apart from typical IT product/system categories [2], like operating systems, data bases, software applications, network equipment, security-related products, chip/RFID cards, *etc.*, quite new, emerging standard applications appear. The intelligent sensors and sensors systems can be considered one of them.

Intelligent microcontroller-based sensors are able not only to measure a physical quantity and to convert it into a signal which can be read by an observer or instrument, but also to process measured values and to send them to other sensors or applications. Usually sensors are organized in the form of sensor systems, based on the network technology, including the wireless technology. With respect to the CC methodology, intelligent sensors can be considered IT products, being a part of a broader IT system, and for this reason the IT inherent risks should be considered for sensors as well.

There are more and more intelligent sensor implementations on the market. Still, the number of completed Common Criteria certification processes of these products has not followed suit. This is typical of emerging CC application domains. Many factors may change this situation. One of them, related to the motivation for this paper, is providing intelligent sensor systems developers with methods, tools and knowledge, helping them effectively use the Common Criteria methodology in their R&D works. The provided method is based on the Common Criteria related IT security design patterns developed and validated in [3,4]. The paper shows that these patterns can be implemented in the Protégé Ontology Editor and Knowledge Acquisition System from Stanford University [5] and demonstrates how to apply these patterns in the IT security development process. The ontology-based model of the IT security development process made with the use of the general purpose Protégé tool as well as experimentations on the model in the intelligent sensors domain are elements of a broader feasibility study to provide input for currently elaborated knowledge-base software supporting IT security developers. Due to different limitations, the basic version of the Protégé tool cannot be used by IT security developers as a tool for end-users. The application of the knowledge engineering methodology should improve the preciseness, consistency and reusability of the CC-compliant IT security development. The paper has an interdisciplinary character and is related to the author’s earlier works dealing with:

- the Common Criteria methodology improvement by introducing the CC-related security design patterns and the IT security development process automation;
- patterns-based intelligent sensors IT security development;
- knowledge engineering methodology application in the IT security domain.

Each of these issues needs a short introduction and a review of the author’s earlier research works with respect to similar research run by other authors.

### 1.1. Common Criteria Methodology and Its Extensions

The reader is encouraged to browse the Common Criteria primer [3](Section 3), extended in [4](Section 2) and supplemented there by the Common Criteria related terms and acronyms. More information about this methodology can be found in [2,6,7]. The Common Criteria (CC) methodology encompasses three main processes:

- the IT security development process, generally leading to the elaboration of the security specification called security target (ST), which defines the TOE built-in security functions;
- the TOE development process, related to the IT product or system development with the use of an assumed technology, including its security functions implementation at the claimed EAL level; the elaborated documentation, later used as evaluation evidences, comprises: the TOE development and guidance documentation, life-cycle definition, configuration management, delivery, flaw remediation, development security, testing, and vulnerability assessment issues;
- the IT security evaluation process, performed by an independent body, leading to certification.

The paper concerns the first process, which consists of the following stages: the security problem definition, security objectives, security requirements and the TOE security functions work-out. The CC standard provides the security functional (SFR) and assurance (SAR) components as the specification means for security requirements. Specification means for other stages, which are called generics, are defined by developers in a different way that causes problems for the common understanding and preciseness of the specification means. The author has introduced semiformal generics, called “enhanced generics”, which do not have this limitation, enabling to create more precise and compact security specifications [7–9]. Enhanced generics, expressing common security features and behaviours, have features similar to those of CC components, allowing parameterization, derivation, refinement and iteration. These works concern the UML/OCL-based IT security development framework (ITSDF) encompassing models of data structures and subprocesses related to the IT security development process. The ITSDF framework was implemented in the computer tool supporting IT security developers. The author’s defined enhanced generics and CC-defined components can be considered the Common Criteria related security design patterns (shortly: “CC-related patterns”), which are discussed in [3,4] in the context of intelligent sensors. Please note that these patterns concern the means used in the security specification building.

### 1.2. Security Design Patterns and Common Criteria Related Security Design Patterns

Engineers from different technology domains, including information technology, elaborate, use and improve their own design patterns, which are considered reusable, proven solutions to problems with respect to a given context. The author’s defined CC-related patterns, focused on the Common Criteria compliant IT security development, are more specific than the commonly used “design patterns” or “security design patterns”. The latter concern IT solutions, including security related solutions.

An extensive discussion of security design patterns can be found in [10]. These architectural patterns for software application concern different issues like: the enterprise security and risk management, identification and authentication, access control models and systems or operating system access control, accounting facilities, firewall architecture, secure Internet applications, IP telephony, and cryptographic key management.

The effectiveness of the use of security patterns with respect to design phases is discussed in [11]. The book [12] presents the UML extension called UMLsec, providing a unified approach to the security features description during the secure IT system development. UMLsec can be used to define different kinds of patterns, e.g.,: secure channel, TLS Internet protocol, electronic purse, secure Java programs, bank applications, biometric authentication systems, electronic signature, *etc.* The patterns defined there can be analyzed with the use of the method provided by [7] with respect to the CC methodology.

The above discussed security patterns, most similar to the patterns introduced in the paper, are related to the security features of IT solutions, especially software architectural solutions. Please note that they do not concern the CC methodology. The patterns presented here can be considered their extensions.

### 1.3. Knowledge Engineering Approach in IT Security Domain

Patterns encapsulate engineering knowledge and experiences, and the specific knowledge is needed to use them to achieve the expected solution of a design problem. A natural way is to use the knowledge engineering methodology to manage design-related knowledge. It allows one to obtain additional advantages offered by the knowledge engineering approach.

Knowledge bases may be elaborated with the use of ontologies. In computer science and information science “an ontology is a formal representation of the knowledge by a set of concepts within a domain and the relationships between those concepts. It is used to reason about the properties of that domain, and may be used to describe the domain” [13]. Ontologies have recently found application in many disciplines where “a common understanding”, “a common taxonomy” or “reasoning” are important, including: web-based applications, medicine, public administration, biology, and information security. Similar needs exist in the IT security development/evaluation domain. For this reason the research on applying the ontology-based method in this domain may bring new advantages.

The presentation of the knowledge engineering principles and the elaboration of ontologies and related knowledge bases is included in the paper [14] and in the documentation available free of charge together with the Protégé tool [5].

The first results of the author’s research in this field were presented in [15], discussing selected issues concerning the Specification Means Ontology (SMO). The paper [16] presents the SMO validation on the firewall example, while [17] concerns its validation on the motion sensor example. SMO was extended to the IT Security Development Ontology (ITSDO), expressing the IT security and TOE development (evidences) processes. ITSDO is equipped with a rather huge number of enhanced generics (*ca.* 350 items). They have general character and can be used for typical IT products or systems.

The experiences gained during the validation show that it would be more convenient to define subsets of generics for specific application domains. The paper is focused on defining and implementing the first of them—the subset for intelligent sensors development, elaborated on the results of the papers [3,4]. The previously elaborated CC-related design patterns will be here implemented as knowledge base items.

The introduced CC-related design patterns base on the specialized security ontology. A short overview of research works on security ontologies will provide suitable background for the presented research and development focused on the ontological approach to the discussed domain.

The first group of reviewed R&D works, most relevant to the presented paper, concern the ontological approach to the Common Criteria (ISO/IEC 15408) implementation:

- the paper [18] discusses an ontological model of the CC functional components mapped to the security objectives with the use of a specialized tool; it concerns only one stage of the IT security development process, omitting others;
- the paper [19] presents an ontological model of the CC assurance components and a query-based tool which supports evaluators in planning the evaluation process, retrieving relevant documents or creating reports; it is focused on the IT security evaluation process only, omitting the IT security development.

The above papers do not consider the entire IT security- and TOE development processes and are not focused on the elaboration of CC-related design patterns specific for some kinds of products or systems, such as intelligent sensors.

Some existing ontology-related research works deal with risk management issues: the work [20] discusses the quantitative risk analysis, the work [21] concerns the ontology-based selection of controls, while the paper [22] focuses on an ontology-centred technology risk management architecture for banking applications. The monograph chapter [23] introduces an ontology and knowledge base related to the advanced security trade-off methods which allow to classify these methods, to order their properties and to help select a right method for the given application.

Some papers are strongly related to the information security management systems, especially those compliant with ISO/IEC 27001 [24], or business continuity management systems compliant with BS 25999 [25].

Additionally, many common security issues ontologies [26–30] are elaborated, specifying different security terms and relationships between them, like: threats, attacks, policies, security of services, security agents, information objects, security algorithms, assurance and credentials, *etc.* A good example of this kind of work is the paper [31], discussing security and trust ontologies which encompass risk analysis issues, security algorithm taxonomy, security functions, attacks and defence, and trust. The number of elaborated security ontologies is growing and they cover all security relevant issues. None of these works is focused on the CC-related security design patterns, especially those for the intelligent sensors applications.

### 1.4. Paper Contents—A Continuation of the Previous Research

The paper is based on the author’s previous research works on IT security of sensors. The work [3] discusses architectures, applications and security issues, including the analysis of threats specific for sensors and their systems. On this basis a generalized intelligent sensor model and, related to it, CC-compliant intelligent sensor security model were elaborated. With respect to these models the CC-related security patterns were defined, expressing basic security features and behaviours of intelligent sensors. The set of patterns was validated on the intelligent medical sensor example and improved on this basis. The work [4] provides an improved notation of patterns and their semantics. Another revision of patterns was done on the basis of the validation on other design example. This time the validation concerned an intelligent methane detecting sensor for mines. Summing up, the works [3,4], allow one to properly define the CC-related security patterns and validate them on varied intelligent sensor projects.

Applying knowledge engineering methods in different application domains usually brings advantages such as better design preciseness, common understanding of terms, improved design reusability and automation, and better project knowledge representation. The paper considers the possibility to achieve these advantages with respect to the Common Criteria application domain.

The paper’s contribution can be expressed as the knowledge engineering methodology application for the intelligent sensors security development. The research and development objectives of the paper are:

- to provide a complete, ontological representation of the CC-related patterns;
- to provide a method to use them as knowledge base items for the security specifications elaboration;
- to evaluate these results on some examples.

The indirect, far-reaching objective is to provide such improved patterns, and the related knowledge how to use them, to a broader group of developers of intelligent sensors and sensor systems.

The Common Criteria IT security development process is complicated, needs specialized knowledge and is poorly supported by specialized software tools. Better support of this process and improvement of its effectiveness with respect to the time and cost are still considered challenges. Different efforts and research works in this area are in progress [32]. The author’s works concern these challenges but are restricted to a specific kind of IT products—intelligent sensors and their systems. The novelty of the paper related to the knowledge engineering methodology application in the Common Criteria standard domain concerns two main elements:

- CC-related patterns for sensors expressed as knowledge base items to specify completely security models for sensors, validated on a real methane detecting sensor in a mine;
- validation of the ontological model of the IT security development process as contribution to develop a professional tool for IT security developers.

The author provides a new category of security design patterns—the CC-related items, defined as knowledge base items, which can be used to specify security properties and behaviours of any intelligent sensor or sensors system. The ontological representation of these patterns creates new possibilities to build security specifications: developers obtain a set of predefined, optimized, sensors-focused specification items supplementing the CC-defined components. Generally, such patterns improve the IT security development process because developers are provided with well defined and ready-made solutions of elementary security issues. The CC-related patterns for intelligent sensors and sensors systems have not been developed yet. They encompass all IT security development stages (not only the selected ones, as presented in [18,19]) and the sensors-related domain is considered one of the emerging applications in the Common Criteria standard. Introducing ontological representation of these patterns gives additional advantages related to better automation and intelligence of this process.

The author’s research aims generally at the elaboration of a software tool to support IT security developers. The paper presents research works on the knowledge engineering methodology application to build such a tool. The research area has an innovative character—so far one knowledge base system for IT security development process has been elaborated [33], however it does not use any ontology. Besides, only two CC-related ontologies were elaborated (Section 1.3, [18,19]) but they represent only selected parts of the IT security development process. The empirical evaluation of the ontology-based model of the entire IT security development process was not performed before. For the empirical evaluation the well known Protégé tool is used. It allows one to create fast prototypes, to check different variants, to assess these variants, to experiment, and to obtain a lot of other useful information on the ontological model properties and behaviour. It is convenient for ontology developers’ experimentations but not for IT security developers as end-users. The aim of this evaluation with the use of Protégé tool, summarized at the end of the paper, is to work out some input to develop a software tool supporting IT security developers [34].

The paper contains two key sections. Section 2 discusses the ontology and knowledge base development process. Section 3 shows some examples presenting the ontology, related knowledge base and their use in the intelligent sensors security development. The additional, last section concludes the applied approach and planned works.

## 2. IT Security Development Ontology (ITSDO)—Version Focused on the Intelligent Sensors Security Development

The IT Security Development Ontology (ITSDO) encompassing security patterns for sensors has been elaborated according to the basic knowledge engineering rules [14] and with the use of the Protégé Ontology Editor and Knowledge Acquisition System developed at Stanford University [5]. ITSDO is expressed by the OWL language, precisely by the OWL-DL (OWL—Web Ontology Language, DL—Description Logics). This OWL version allows automatic reasoning. The iterative, top-down ontology development process encompasses steps presented shortly in Subsection 2.1 through 2.7. Selected results of this process are shown in Section 3. The paper presents the ITSDO version that was slightly improved with respect to the validation on intelligent sensors (the range of improvements: [4]/Subsection 3.2).

### 2.1. Definition of the Ontology Domain and Scope, and Competency Questions

The domain of the ITSDO ontology is constituted by the Common Criteria compliant IT security development and the TOE development processes, but the paper concerns the former process only and focuses on the security patterns for sensors. ITSDO provides common taxonomy for specified items, allowing one to better understand them and better express relationships between them. Important issues concerning the ontology domain are competency questions [14] which are defined as questions that the ontology related knowledge base is able to answer. These answers define the scope of the ontology. Please note that ontologies and their knowledge bases are developed incrementally, and after exceeding “a critical mass” a knowledge base allows to get answers to more and more advanced questions. The ITSDO knowledge base prototype is able to provide answers to basic questions related to the security development of any IT product or system though examples presented in the paper are focused on sensors.

**Figure 1.**
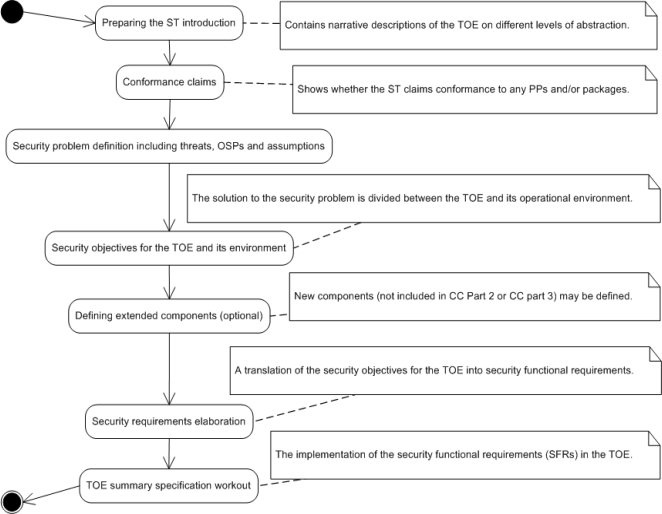
The CC-compliant IT security development process as a UML activity diagram.

The UML activity diagram shown in Figure 1 presents particular stages (subprocesses) of the CC-compliant IT security development process. The research is focused on the four most complicated stages where the ontological approach can be most suitable, *i.e*., the security problem definition, security objectives, security requirements and the TOE security functions work-out. The key requirements for the ITSDO ontology domain are:

1. The ITSDO ontology should be able to express the entire IT security development process aimed at the elaboration of the security target (ST) specification for any IT product or system (TOE), *i.e*., it encompasses the entire Common Criteria applications domain. ITSDO includes the ST hierarchical structure, expressing its parts and relations between them, e.g., the security problem definition, security objectives, security functional and assurance requirements, and of course their parts, e.g., threats, OSPs and assumptions as the security problem definition (SPD) parts, *etc.* These “parts” play the role of containers for security-related data for different IT products or systems. It implies the need to define the second group of terms used as the specification means for these containers;
2. The ITSDO ontology should be able to express the specification means for a given family of IT products or systems, here: “the intelligent sensors and sensors systems”. The Common Criteria specification means include: general-purpose functional and assurance components (defined in the CC) and TOE security specific generics: for threats, security policies, assumptions, objectives and functions description (*i.e*., the here discussed CC-related patterns for intelligent sensors and systems);
3. The ontology should express two kinds of relationships existing in the IT security model of an IT product or system. The vertical relationships express relations between particular ST parts (containers) and specification means, placed into these containers. For example, we can put threat-type generics as specification means into the threats specification, which is considered a container. The horizontal ones express relations between specification means belonging to the neighbour levels of the security model, e.g., between any threat-type generic and a security objective-type generic which counters it;
4. The ITSDO ontology should be able to demonstrate generally the ability to retrieve knowledge within the domain to assess the needs and expectations of IT security developers with respect to this issue, mostly the ability to retrieve all relationships within the project between any elementary security problem (threat, OSP, assumption) and its solutions, refined step by step—starting from security objectives, through functional requirements, to security functions implementing the requirements (and to evidences showing their implementation—not discussed in this paper). Retrieving may also concern finding some items of given characteristics to solve any security issue or to refine a given security issue. The retrieving allows to compare different solutions or their parts, helps to assess the completeness of solutions or find redundant solutions, *etc.* Generally, the retrieving should be able to support IT security developers in typical activities;
5. The ITSDO ontology should be able to implement the reasoning possibility in the future to research IT security developers’ needs and expectations with respect to this issue. To ensure this and to make the elaborated models more precise in the future, the OWL-DL is preferred;
6. ITSDO work-out should allow:
  - experimentations on the model of the IT security development process, on sensors example—to identify the basic concepts concerning this process and the relations between them.
  - the elaboration of the ontological model of the sensor/sensors systems domain, its validation in the sense of an adequate ontological representation of this domain, and finally its application in the elaborated software tool supporting sensors systems developers.
7. ITSDO will be implemented in the Protégé environment;
8. The self explanatory names of classes, properties and instances allow developers (humans) to better understand the terms during experimentations on security models.

### 2.2. Considering the Possible Reuse of Existing Ontologies

Reusing (integrating) the existing ontologies into the created one can speed up the ontology development process, can extend the ontology scope and the range of its applications. During the review of the paper-related R&D works (Section 1.3) two reusable ontologies [18,19] were encountered. The integration may be restricted to the Common Criteria components only, but reusing cannot be considered here because these ontologies are not publicly available.

### 2.3. Identifying Important Terms in the Ontology

The basic set of important terms encompassed by the ITSDO ontology was identified during the ontology domain analysis performed while the UML/OCL models of the ITSDF framework were elaborated [7]. With respect to the intelligent sensors domain of application the basic terms expressing threats, security policy rules, assumptions, security objectives and functions are identified in the papers [3,4]. The identified terms are used here to define the ontology classes and their properties.

### 2.4. Definition of Classes (Concepts) and the Class Hierarchy

ITSDO is developed iteratively with the use of a typical top-down approach with certain bottom-up activities. The right knowledge representation, as the sensors security patterns, requires the analyses of terms and relations between terms to properly express such relations as: class-instance, class-subclass and class-superclass, and to decide what should be expressed by a class and what by a property, what by abstract classes and what by classes which have instances [14]. While elaborating the ontology class hierarchy, the possibility of its future evolution should be considered. Most class names are self explanatory, though their lengths should be limited.

### 2.5. Definition of Class Properties and Their Restrictions

Classes can have some properties assigned. Three kinds of properties can be distinguished [14]:

- object-type properties (also called “instance-type properties”) are used to express “complex properties”, *i.e*., relationships between an instance of the given class (the object) and other instances; the two following situations are typical: the first one occurs when the given instance consists of other instances, the second one when the instance points to other instances; an instance-type property is attached to the classes which are called a domain; the classes indicated by this property are called a range;
- data-type properties are used to express “simple properties” or “attributes”, *i.e*., intrinsic or extrinsic properties of the instances of the most elementary classes; these properties are expressed by data types commonly used in modelling or programming, e.g.,: integer, byte, float, time, date, enumeration, string, *etc.*;
- annotation, RDF-based (RDF means Resource Description Framework) properties, are used to document different ontology items (classes, properties, instances); the most known example of this property is the *rdfs:comment* property which gives more explanation of the given ontology item.

For properties, the restrictions can be defined. They specify or limit a set of possible values for the given property. The ITSDO ontology uses all types of properties (it will be exemplified in Section 3). The defined properties are also self-explanatory. The properties contents expresses the features of the sensors design patterns.

### 2.6. Creating Instances and Filling in Their Properties

Instances represent non-abstract class members. For ITSDO the instances are defined mainly for the classes of the lowest hierarchy level. The instances with filled-in properties represent the key Common Criteria methodology artefacts, like CC components, CC-related design patterns and evaluation evidences. The instances with their properties expressing relationships constitute the ITSDO-related knowledge base. Some auxiliary instances of abstract meaning are used to organize the knowledge base. The instances of classes representing threats, security policy rules (OSPs), assumptions, security objectives, and functions represent sensors-specific design patterns. The instances of the functional and assurance CC components have general meaning and can be used for any IT products or systems.

### 2.7. Testing and Validation of the Developed Ontology

During its elaboration, the ITSDO ontology was tested—to avoid commonly known errors discussed in [14], and validated—to assess its usability. The Protégé environment provides some facilities to support these experiments. Testing is done with the use of menu options: “Checking consistency” and “Run ontology tests”. Certain tests are performed manually in the form of ontology inspections supported by OWLViz—a built-in visualization tool (plug-in). During the validation process the user checks if the right structures of instances are composed, if they have assumed properties, if the needed information can be retrieved properly by queries from the knowledge database, and if the forms are properly defined. The ITSDO ontology validation encompasses the basic scenarios of the ontology use.

## 3. Knowledge Base Presentation and Use in the Intelligent Sensors Security Development

The developed ontology and related knowledge base need validation. The paper presents selected experiments which should get the answers, whether:

- the CC-related design patterns are enough to fully specify security features and behaviour of sensors with respect to the Common Criteria methodology.
- the knowledge encompassed by this specification can be managed, *i.e*., introduced, modified, replaced, retrieved, *etc.*

The selected issues of the ontology validation process will be exemplified by the Protégé tool and discussed in [4] the Methane Early Detection Intelligent Sensor (shortly: MEDIS). From the point of view of the Common Criteria methodology this sensor is considered the TOE (target of evaluation). IT security developers should elaborate a security target document and evaluation evidences for the TOE.

Example 1. Knowledge base representation of the MEDIS security target.

**Figure 2.**
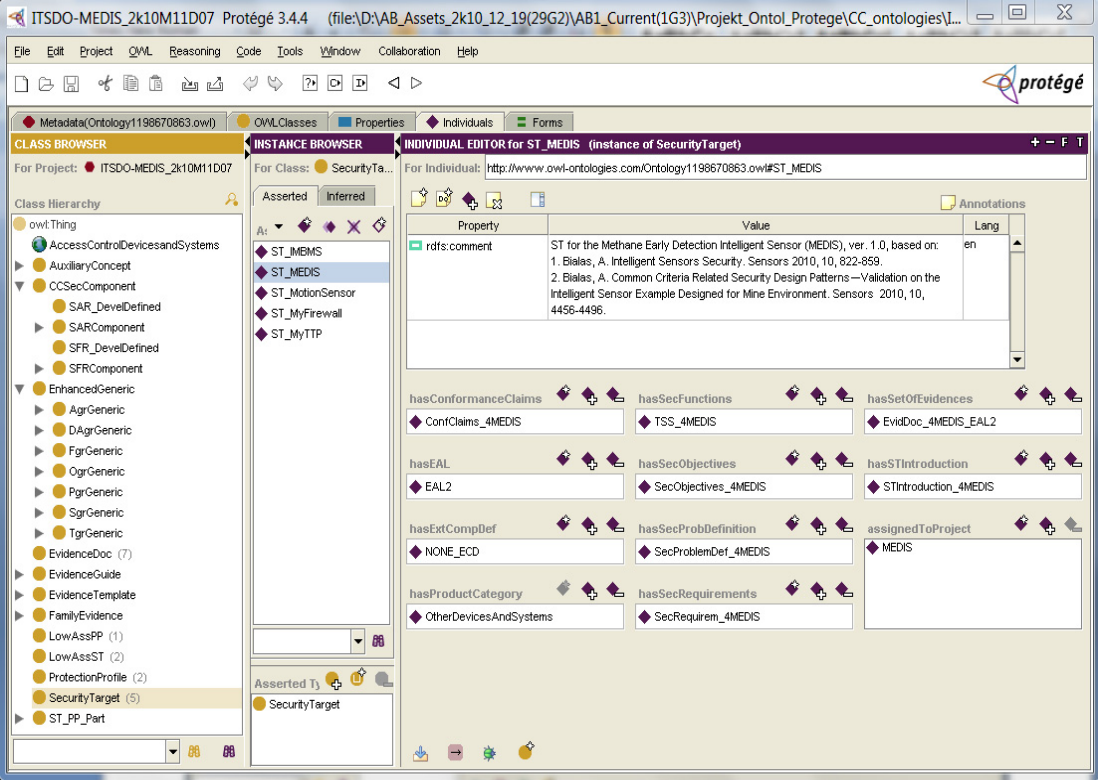
The MEDIS security target viewed in the Protégé tool [5].

The Protégé tool is designed to develop and manage ontologies and related knowledge bases of different application domains. One of the ontology examples is the ITSDO ontology developed by the author and related to the Common Criteria methodology. Figure 2 presents three important panels of the Protégé tool. On the left, the Class Browser shows the class hierarchy. The ITSDO classes are subclasses of the basic OWL class called *Thing*. The main ITSDO classes are:

- *AuxiliaryConcept*, used for ontology organization;
- *CCSecComponent*, representing the CC-defined security assurance- [1]/part 3 and security functional [1]/part 2 components; they are used as specification means patterns for security requirements placed in the security targets and protection profiles structures;
- *EnhancedGeneric*, representing the author’s defined patterns for assets (*DAgrGeneric*), subjects (*SgrGeneric*), threats (*TgrGeneric*), policies (*PgrGeneric*), assumptions (*AgrGeneric*), objectives (*OgrGeneric*), and functions (*FgrGeneric*) [3,4];
- *SecurityTarget*, *ProtectionProfile*, *LowAssST*, *LowAssP*, expressing the patterns of the CC-defined documents of security requirements [1]/appendices in the part 1;
- *ST_PP_Part*, expressing parts of the above mentioned documents of security requirements;
- *EvidenceDoc*, *EvidenceGuide*, *EvidenceTemplate*, concerning evaluation evidences; generally *EvidenceDoc* expresses the TOE evaluation evidences elaborated with the use of patterns (*EvidenceTemplate*) and methods (*EvidenceGuide*); this kind of evaluation documents patterns [35,36] is not discussed in this paper.

Please note the classes hierarchy, *i.e.*, classes having subclasses that can be viewed when clicking on the triangle symbol.

For the highlighted *SecurityTarget* class its instances (marked as rhombuses) are shown in the middle panel (the Instance Browser). One of the five security target instances is the considered *ST_MEDIS* security target. For this selected instance its details are shown in the right panel (Individual Editor panel; please note that Protégé uses the older term “individual” instead of the OWL term “instance”). Each part of the security target is expressed by one object property value, being an instance. Apart from the parts requested by the CC standard, additional information about the product category, project, claimed EAL and evidences is also expressed by object-type properties. In the upper part of the panel the annotation property (please note: *rdfs:comment*) presents auxiliary information about the MEDIS project and the related security target.

Example 1 shows how the CC basic security specification, *i.e*., security target, can be precisely expressed with the use of the knowledge engineering approach. Using the predefined ST structure (that can be considered the evaluation document pattern) the IT security developer creates the security target instance and its parts for different TOEs, including the MEDIS sensor. Each part has been refined according to the top-down methodology. The ST introduction is rather trivial (textual description of the TOE and its ST). The first important section elaborated by the developer is the security problem definition (SPD). This example has general character—in the same way any security target, not only that of sensors, can be expressed.

Example 2. The security problem definition and solution.

**Figure 3.**
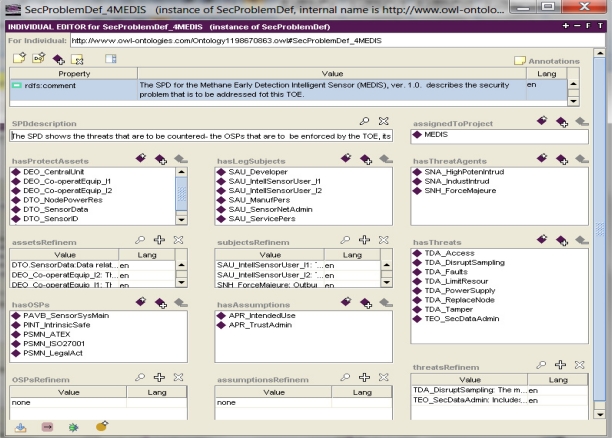
The MEDIS security problem definition in the Protégé tool [5].

Please note that the ontological top-down model considers all details of the given ST part (Figure 2). By clicking on any instance, its details, also represented by properties, can be observed. For example, by clicking on the *SecProblemDef_4MEDIS* instance (Figure 2), the security problem definition for the MEDIS sensor is displayed in a pop-up window of the Protégé tool (Figure 3).

The SPD includes its general description, placed as a text within the *SPDdescription* data property. Please note the specification of assets (the *hasProtectAssets* object property with multiple values allowed) and refinements of some assets (please note the *assetsRefinem* data property). Particular items within this property represent design patterns specifying different examples of the sensors related assets. Similarly, other enhanced generics, as knowledge base items (patterns), representing legal subjects, threat agents, assumptions, OSPs (Organizational Security Policies), and threats are placed in the right properties.

For example:

- the asset pattern *DTO_SensorData* expresses all data sampled and processed by the MEDIS sensor;
- the subject pattern *SNA_HighPotenIntrud* represents an attacker having high level skills, enough resources and deep motivation to perform a deliberate attack against MEDIS and the sensor system where this sensor works;
- the threat pattern *TDA.DisruptSampling* concerns the situation where “Users or intruders could try to manipulate the sensor input causing wrong input data”.

Please note that patterns expressed as ontology items (instances) comply with their traditional definitions (as enhanced generics) presented in the paper [4].

The entire SPD, as an ontology item, is annotated by its *rdfs:comments* property. The IT security developer analyzes the security problem and specifies it using enhanced generics expressed as knowledge base items (CC-related design patterns).

The SPD problem is solved by specifying security objectives for the TOE and/or for its operational environment. The IT security developer analyzes particular threats, OSPs and assumptions and assigns to each of them one or more security objectives for the TOE and/or its environment using enhanced generics, *i.e*., CC-related design patterns.

**Figure 4.**
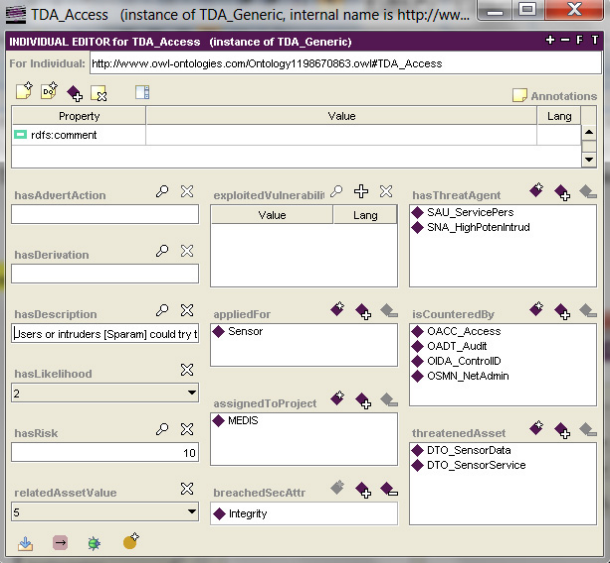
The threat representation in the knowledge base [5].

One of the analyzed threat examples, *TDA_Access*, is shown in Figure 4. This pop-up window is displayed by clicking on the *TDA_Access* instance from the *hasThreats* property (Figure 3). The threat *TDA_Access* represents scenarios where “Users or intruders could try to access sensor functions or data they are not allowed to”.

The IT security developer analyzes the meaning of the threat (the *hasDescription* property), threatened assets (sensor data and services) and possibilities of threat agents (attack potential), exploited vulnerabilities, and finally assigns some security objectives using the *isCounteredBy* property:

- providing sensors with properly managed unique identifiers (*OIDA_ControlID*);
- controlling by means of sensors the access of connected entities (*OACC_Access*),
- auditing any attempts to undermine the sensor security and tracing them to the associated entities (*OADT_Audit*);
- implementing procedures of sensor network administration and security policy (*OSMN_NetAdmin*).

Assigning one or more security objectives is identified as the solution of the elementary security problem. Additionally, a simple risk analysis is possible which is made to compare different threats with respect to the event likelihood and the threatened asset value, both assessed with the use of the predefined enumerative scales. Assuming that in this project the event likelihood and the related asset values (both assets considered together) are assessed in the range: 0–5, the risk as their product is measured in the range: 0–25. The risk concerning *TDA_Access* is 10/25 (40% of the maximum value in this project). This simple risk assessment facility allows to order threats by risk, and this ordering gives useful input for the applications of measures by assigning security objectives.

Example 3. Specifying the security objectives for the MEDIS sensor.

**Figure 5.**
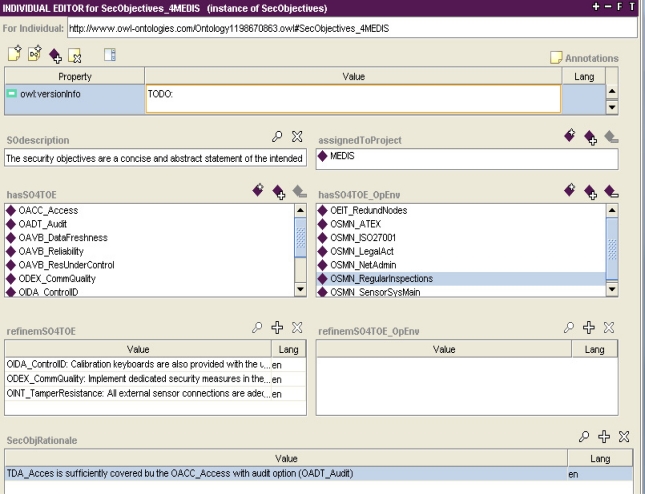
The MEDIS security objectives in the Protégé tool [5].

Solving fully the SPD, a complete set of security objectives is reached, as it is shown in Figure 5. Security objectives represent safeguards built into the TOE (the *hasSO4TOE* multiple-valued property) or applied within the TOE operational environment (the *hasSO4TOE_OpEnv* multiple-valued property). In the next stage of the IT security development the first group will be expressed by the security functional requirements (SFRs) and implemented later within the TOE security functions. The second group will be refined in different documents (evaluation evidences provided for the IT security evaluation/certification process–not discussed here).

The *SecObjectives_4MEDIS* instance (Figure 5) expresses these both groups of objectives. As usual, some of them are refined to better express the project needs. The security objectives should be justified as a whole (the *SecObjRationale* property)–the justification has not been finalized yet by the IT security developer in this project.

Example 4. Specifying security requirements for the MEDIS sensor.

The security objectives for the TOE should be expressed in a unified, semiformal way with the use of CC-defined functional components [1]. To obtain the security functional requirements specification, each TOE security objective is analyzed by the developer and one or more security functional components are assigned to it. This analysis is performed within a certain context defined by the SPD elements and the risk. At this stage of the IT security development, the security functional components play the role of CC-related security patterns.

**Figure 6.**
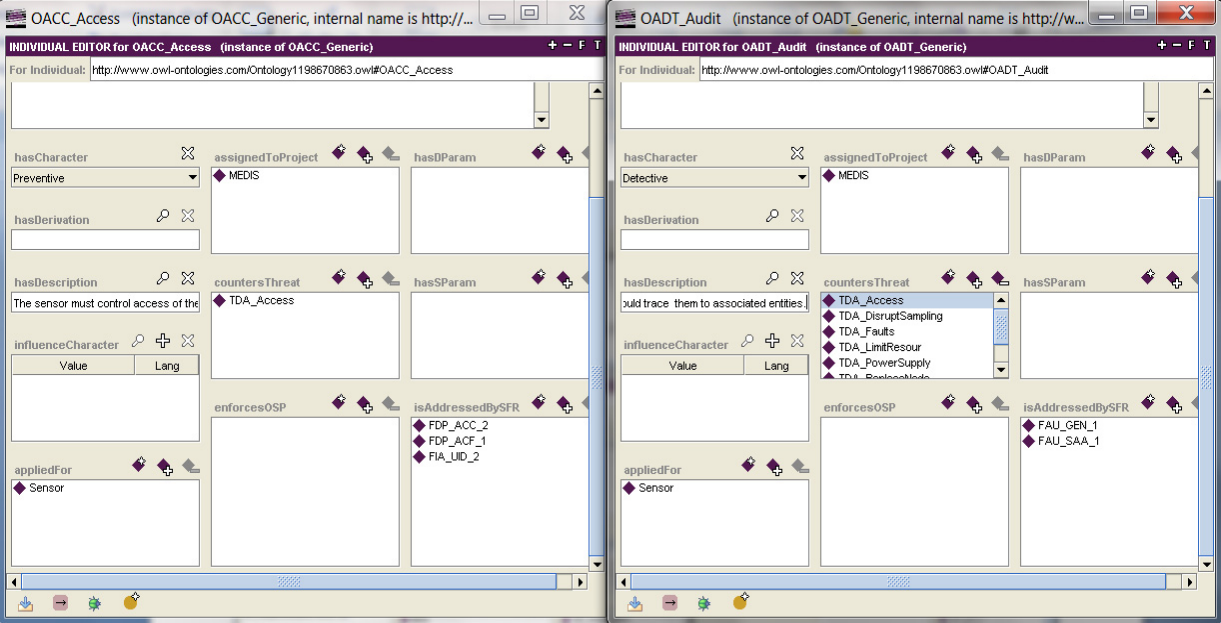
Specifying the functional security requirements for the security objectives using the tool [5].

Figure 6 shows two aforementioned instances of security objectives countering the *TDA_Access* threat and the security components assigned to them. *OACC_Access* is represented by components responsible for the user identification (*FIA_UID_2*) and for defining and executing the access control policy (*FDP_ACF_2*, *FDP_ACC_2*) [1](part 2). The *OADT_Audit* security objective, supporting *OACC_Access,* is represented by components responsible for the audit data generation (e.g., when access is violated) and audit data analysis (*FAU_GEN_1*, *FAU_SAA_1*) [1](part 2).

Example 5. Specifying the security functions which implement functional requirements.

Analyzing each TOE security objective, the IT security developer expresses, step by step, the entire security functional requirements specification with the use of patterns. Here functional components play the role of patterns. The Common Criteria standard requires to perform the rationale of this specification as well.

**Figure 7.**
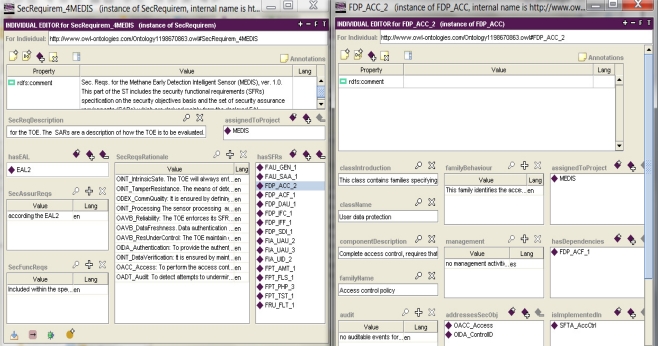
The security functional requirements and their particular components.

The left part of Figure 7 presents a complete set of security requirements for the MEDIS sensor. The security assurance requirements are specified by declaring the Evaluation Assurance Level (EAL), here: *EAL2*. Please note that the given EAL implies the proper package (set) of SARs [1](part 3). The right part of this figure presents the *FDP_ACC_2* components details. Please note the security objectives which address these components (*OACC_Access*, *OIDA_ControlID*) and the *SFTA_AccCtrl* security function which implements them (pointed by the *isImplementedIn* property).

Example 6. Finalizing the IT security development process.

The IT security development process is finalized by the TOE summary specification (TSS) work-out. This specification includes all security functions needed to implement the security functional requirements on the assumed EAL level. For this stage the patterns were defined as well, though they are used rather as examples to define specific functions meeting IT product/system requirements [4].

**Figure 8.**
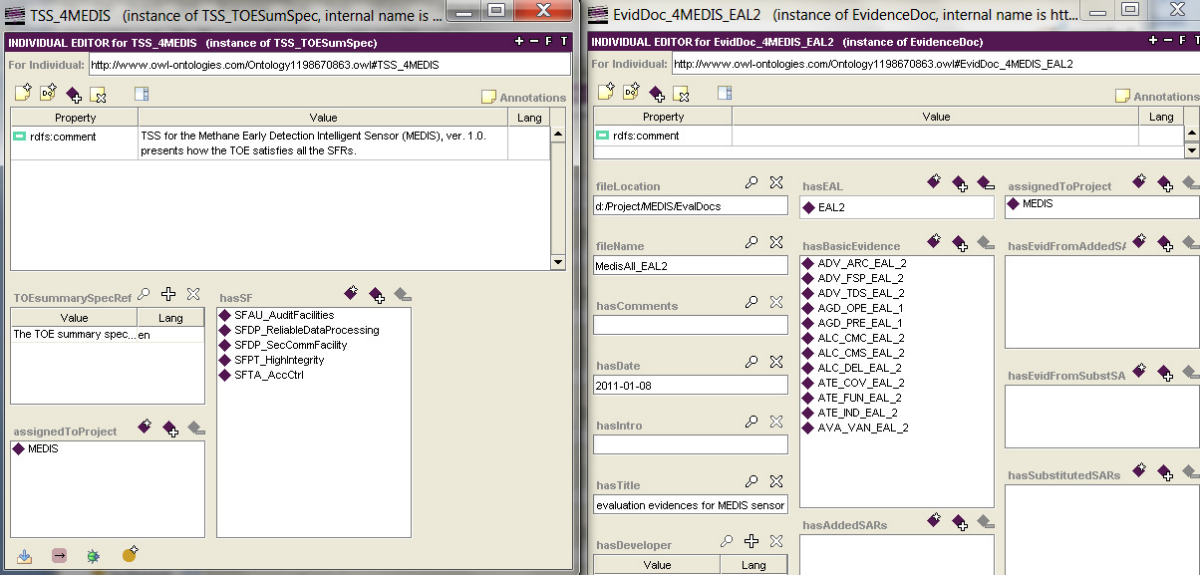
Specifying security functions and evidences [5].

The left part of Figure 8 presents the TSS (the *TSS_4MEDIS* instance) for the considered MEDIS sensor, *i.e*., functions (pointed by the *hasSF* property) responsible for access control, reliable data processing, secure communications, ensuring robustness and providing audit facilities for the MEDIS sensor. These functions will be implemented on the EAL2 level of assurance. The right part of Figure 8 presents general specification of evaluation evidences (the *hasBacicEvidence* property). Issues dealing with evidences are not discussed in the paper.

Please note that only basic evaluation evidences are specified (it means: no additions, no substitutions are presumed for the MEDIS [1](part 3)). Evaluation evidences concern: architecture, interfaces, decomposition, guidance documentation, configuration management, delivery procedures, testing, and vulnerability assessment—all of them on the EAL 2 level assurance components.

Particular evidences, identified as instances, are described in separate documentations whose structures and contents are the subject of the project [34].

Example 7. Knowledge retrieving and analyzing during the IT security development process.

The developed CC-related design patterns placed as the knowledge base items allow to issue security specifications of different IT products or systems.

These items, and relations between them, represent the project knowledge. The project data elaborated on the patterns basis can be easily modified or reused for other projects. The relations between project items can be analyzed with the use of a simple query option of the Protégé tool.

**Figure 9.**
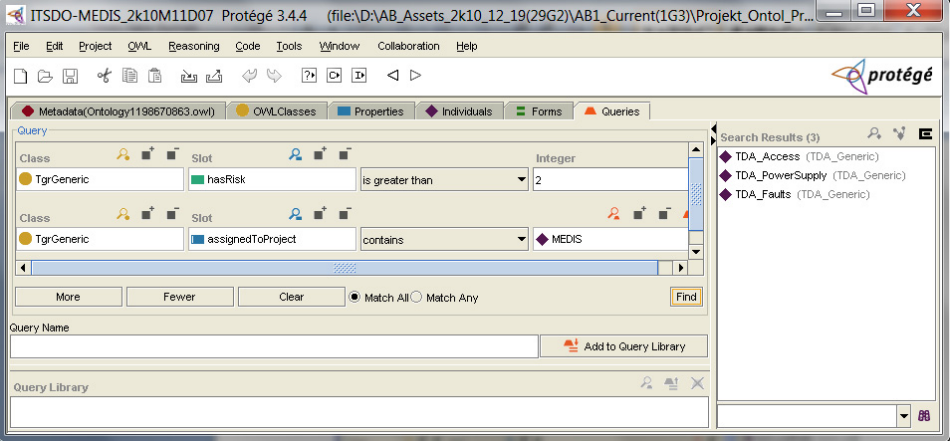
Project knowledge retrieving with the use of a simple query tool [5].

Let us assume that the IT security developer looks for threats with the risk value higher than 2 in the predefined scale. Figure 9 shows the applied query example retrieving 3 threat cases which satisfy this condition. Next, the developer intents to analyze one of them, e.g., *TDA_Access*, asking about security objectives countering this threat.

**Figure 10.**
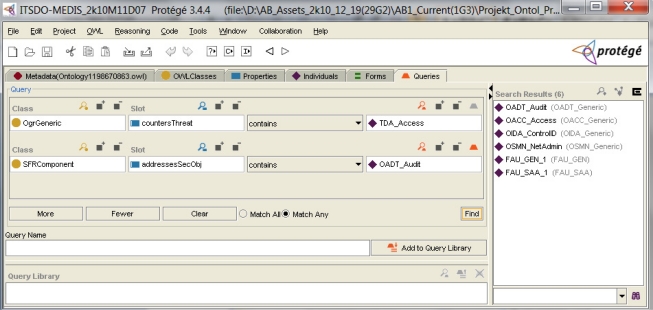
Project knowledge retrieving with the use of a simple query tool [5]—a continuation.

In Figure 10 the first query line retrieves these four objectives. One of them is *OADT_Audit*. Asking about functional components related to the audit, the developer adds the second line of the query (using the option “Match Any”) and obtains two components: *FAU_GEN_1* and *FAU_SAA_1*. The queries can be stored and managed by the Protégé Query Library. They can be organized hierarchically—a query can search for items within the results of other query. The query facility is rather simple and may not satisfy all IT security developers.

## 4. Conclusions

The paper concludes research on a special kind of security patterns designed to elaborate the Common Criteria compliant security specifications for intelligent sensors. Earlier papers [3,4] defined and validated these patterns on real examples of sensors, while this work is focused on the ontological representation of these patterns to achieve extra possibilities and advantages offered by the knowledge engineering methods. The validation presented in the paper ought to provide an answer if these advantages are achieved.

At the beginning of the paper, the research background was discussed. Then the elaboration of the ontology and the related knowledge base dealing with the paper contribution were concisely presented. This elaboration is based on the commonly used Protégé tool. The key section of the paper concerns the exemplification of the created ontology on the IT security development process.

### 4.1. Summary of Validation Example

The Methane Early Detection Intelligent Sensor (MEDIS), discussed in the paper [4], was chosen as the object of validation. The reader can compare both variants of the applied IT security development process:

- performed manually, but patterns-based, described in [4], and,
- the ontology-based variant, discussed here

The presented validation shows how the key security issues can be expressed using the ontological representation of the CC-related security patterns. During the validation, the MEDIS security target was developed step by step. The security problem definition (SPD) requires to identify the threats, organizational security policies and assumptions for the TOE. In the same way, the method proposed here allows to specify the active entities (legal subjects, threat agents) and passive entities (protectedand threatened assets). Both these entities can be used as parameters values for the SPD items. For each elementary security issue its elementary solution in the shape of a security objective was formulated. Then particular security objectives were transformed into security functional components. Their implementation, in the form of technology-dependent security functions, was shown as well.

All these issues were specified precisely and concisely with the patterns defined as the ITSDO knowledge base items (instances) and the relations between these items (properties). The basic examples of these relationships are:

- the parameter and the value substituted to it; for example, most of the threat patterns have parameters: *Sparam* [4], representing a threat agent and *Dparam* [4], representing a threatened asset; the given threat pattern expresses a certain group of similar malicious behaviours (attack methods); using the threat pattern, the developer can specify the concrete threat case by substituting these parameters with concrete subjects and/or assets and by adding optionally the TOE-specific details as the threat refinement; the parameter value substitution is implemented with the use of an object type property (Figure 4, the *hasThreatAgent* and *threatenedAsset* properties); refinement is expressed, for example, by a data property (Figure 3, *threatsRefinem*);
- the elementary solution to the elementary security problem; it may concern the assignment of one or more security objectives (for the TOE and/or for the TOE operational environment) to any threat, OSP and/or assumption; it is implemented by non-functional (*i.e*., having multiple values possible) object properties (Figure 4, the *isCounteredBy* property, Figure 6, the *countersThreat* property);
- transforming TOE security objectives into security functional requirements (Figure 6, the *isAddressedBySFR* property), as well as the implementation of the SFR within the TOE security functions (Figure 7, *isImplementedIn*) are very similar issues to the above mentioned mapping of the elementary solution to the elementary security problem;
- miscellaneous relations example, like: assigning the EAL level to the TOE design, assigning the implied evidences to the claimed EAL, expressing the security assurance requirements (components) of the given EAL package, and many others.

The data properties are as useful as the object properties. The data properties are used especially to assign textual descriptors to the main ontology items. The refinement operation is based on this property type too (Figures 3 and 5). The MEDIS security target does not use iteration though this operation is possible. The iteration is carried out by placing into the security specification many instances of the same pattern with different parameter values substituted and/or with different refinements.

The validation, focused on the MEDIS security target, concludes that all issues of this security target can be expressed with the knowledge base items derived from the ITSDO ontology. Generally, two IT security development processes variants, *i.e*., the one based on patterns [4] and the other based on the ontological representation of the patterns, are convergent, but the latter one offers extra advantages related to the ontological approach discussed in the next subsection. The ontological approach allows to elaborate the conceptual security model of the sensor with the use of patterns and relations between them. This model facilitates security analyses supporting the security target work-out.

The ITSDO ontology was shown from the ontology development tool perspective. The readers can see domain concepts and relations through the rather friendly GUI (Graphical User Interface) of this tool. It is good for the experimentations on ontologies (different variants can be checked) but it cannot be used directly by the sensors and security developers (uncontrolled data change possibility). In reality the tool is based on the formal specification of the ontology expressed in the OWL language. To exemplify the ITSDO ontology representation, the paper is supplemented by two simple examples. The readers should be familiar with the basics of the XML, RDF, RDFS (RDF Schema) and OWL notations.

Example 8. The ontology *TEO_Generic* class representation (please refer to Figure 2, The Class Browser).

A part of the ITSDO ontology presenting the *TEO_Generic* class is listed below (starting from the <owl:Class rdf:ID=“TEO_Generic”> tag, ending at the </owl:Class> tag). This class is a subclass of *TgrGeneric*, representing any kind of threat. The class has 2 comments assigned, the first one says that it concerns attacks against the TOE environment, while the second one that formerly this class was called “TIT”. Further we can see that this class is disjointed with the *TES_Generic* and the *TDA_Generic*, described elsewhere.

<owl:Class rdf:ID=“TEO_Generic”>
  <rdfs:subClassOf>
  <owl:Class rdf:ID=“TgrGeneric”/>
  </rdfs:subClassOf>
  <rdfs:comment rdf:datatype=http://www.w3.org/2001/XMLSchema#string
>Threats against the TOE operational environment</rdfs:comment>
  <rdfs:comment rdf:datatype=“http://www.w3.org/2001/XMLSchema#string”
>former TIT</rdfs:comment>
  <owl:disjointWith>
  <owl:Class rdf:ID=“TES_Generic”/>
  </owl:disjointWith>
  <owl:disjointWith>
  <owl:Class rdf:ID=“TDA_Generic”/>
  </owl:disjointWith>
</owl:Class>

Example 9. The *hasThreatAgent* object property representation (please refer to Figure 4 to see this property).

Please note that this property expresses the relation between any threat (the *TgrGeneric* class—a domain) and its threat agent, being any subject-type generic (the *SgrGeneric* class—a range). This relation is inverse to the *usedAsThreatAgent* relation. The discussed relation belongs to a broader group of relations called *objects4SpecMeans*.

<owl:ObjectProperty rdf:about=“#hasThreatAgent”>
<rdfs:domain rdf:resource=“#TgrGeneric”/>
<rdfs:range rdf:resource=“#SgrGeneric”/>
<owl:inverseOf rdf:resource=“#usedAsThreatAgent”/>
<rdfs:subPropertyOf rdf:resource=“#objects4SpecMeans”/>
</owl:ObjectProperty>

### 4.2. Summary Concerning the Elaborated Ontology

The paper presents multidisciplinary research and development works encompassing mainly the security engineering and knowledge engineering domains. The paper presents works focused on the sensor and sensors system security development. The motivation of the paper was to improve the CC-compliant IT security development by applying the knowledge engineering methodology and to support the Common Criteria application in the intelligent sensors development domain, including collaborative sensors applications.

The mentioned improvement may concern the preciseness, consistency and reusability of the CC-compliant IT security development, better design knowledge management and providing this knowledge for developers. All of them are related to the typical advantages brought by the ontological approach [14]:

1. The elaborated ITSDO ontology, in the same way as many others, enables common understanding of the terms in the ontology domain, *i.e*., terms related to the Common Criteria methodology. The paper concerns the IT security development process only, though this ontology is broader and encompasses the evidences and their elaboration as well as management. With respect to the IT security development process, ITSDO defines precisely the security specification structures and the specification means represented here by the CC related security patterns. The relationships between these patterns, like: “counters”, “enforces”, “upholds”, “addresses”, ”implements”, *etc.* are important too. This common understanding of the structure of information concerns mostly people (*i.e*., IT security engineers, evaluators, users and other stakeholders, called CC consumers).
2. Generally, ontologies facilitate the reuse of domain knowledge. In the considered case, the CC related security patterns (CC—defined components and author’s defined enhanced generics) can be used for other sensors projects. The ITSDO knowledge base can be considered a library of predefined patterns for sensor and sensor system applications.
3. ITSDO, similarly to many other ontologies, allows to make explicit assumptions for a domain. It deals mainly with predefined relationships facilitating the security target development, e.g.: mapping a typical solution to the given elementary security problem, mapping typical security functional requirements (SFRs) to the given security objective, proposed implementation of the given SARs by the security function. The predefined relationships are based on the experience gained with previous projects. The developer can use these relationships or can add new ones. These assumptions made with respect to the intelligent sensors security features can be considered an important kind of design knowledge. Generally, the IT security development process is supported by disseminating this experiences-based knowledge to other developers.
4. The considered ontology is able to separate domain knowledge (items related to specification means as a whole, designed to use in many different projects) from operational knowledge (how to use this domain knowledge to compose a new security target or protection profile document). It facilitates the automation of the most repeatable activities of the IT security development process.
5. ITSDO, as many other ontologies, supports the analyses of the domain knowledge, like supporting a simple risk analysis to consider some variants of security solutions. Generally, it facilitates the automation of the most difficult activities of the IT security development process, though this issue needs further research.

### 4.3. Future Development and Implementation

The ITSDO ontology and related knowledge base are still under development. This is an iterative and incremental process, beginning with the basic concepts and properties. As more complicated concepts, properties and restrictions are added, the ontology is getting more matured and is able to express more sophisticated relationships and to get answers to more advanced competency questions. These initial efforts are considerable but further automation effects, reusability and preciseness, balance these efforts. Currently the R&D activities are focused on:

- the extension of the reasoning capabilities allowing more sophisticated competency questions;
- the elaboration of subsets of patterns for domains of application other than sensors;
- the ontological representation of the evaluation evidences patterns, concerning evidences elaborated on the ST basis during the IT product or system (TOE) development and provided for the IT security evaluation process.

The validation of the IT security development process ontology-based model with the use of the Protégé tool and the intelligent sensors security domain shows that:

- all advantages and possibilities offered by the ontological approach, summarized in this section, may be achieved with respect to the IT security development process;
- the ontology-based model of the IT security development process fully expresses the developers’ needs and expectations (precise modelling of the complex domain, possibility to elaborate the security target for a given sensor completely, retrieving data to build the TOE security model, reusability); although the ontology encompasses the entire development process and sufficient specification means, the reasoning has not been developed yet but it will be possible in the next version thanks to OWL-DL; requirements related to the reasoning need further research.

The elaboration of the ontology-based model of the IT security development process with the use of the general purpose Protégé tool, along with the experimentation on the model and the intelligent sensors domain, were elements of the feasibility study allowing to define assumptions for the currently elaborated knowledge-base software supporting IT security developers. The readers, such as sensors developers and security developers who are not familiar with the knowledge engineering basics, get an exemplification of their well known security issues in the environment which is quite new for them.

The experimentations with security ontologies are based on the Protégé 3.x version, because it was stable when the experimentation started and it had sufficient possibilities for this purpose. Other OWL-based tools can be used as well, like: Protégé 4.x [5], Semantics works [37], NeOn Toolkit [38], TopBraid Composer [39].

The ITSDO ontology was developed using the tutorial [14] and other documentations of the Protégé community, like [40]. The author wants to point out other references concerning the ontology development, e.g., Staab *et al.* [41] (the formal basis, tools, approaches in different ontology application domains), W3C consortium documents concerning the semantic web [42] (standards, drafts) and many other publicly available papers and guides on the above mentioned ontology-related tools.

Currently the ITSDO ontology is used as a conceptual model for the CCMODE (Common Criteria compliant, Modular, Open IT security Development Environment) R&D project [34]. The objective of the project, co-financed by the EU within the European Fund of Regional Development, is to work out a CC-compliant methodology and tools to develop and manage development environments of IT security-enhanced products and systems for the purposes of their future certification. The basic products of the CCMODE project will be the following: knowledge, patterns (including documentation, procedures, evidences, specification means, *etc.*), methodology and tools used to create and manage development environments by different business organizations. The IT products and systems developed in these environments, having measurable assurance (EAL), can be certified and used in high risk applications.

The CCMODE project assumptions and first achieved results were presented at the 11th International Common Criteria Conference [35]. Providing IT security developers with the Common Criteria related security design patterns and knowledge how to use them allows to:

- facilitate and speed up the IT security development process;
- improve the quality of evidences, because they are more consistent and include all details required by the considered assurance components and best practices;
- computer support of the IT security development process.

The results of the R&D on ITSDO are used in the CCMODE project, especially to elaborate knowledge engines, evaluation evidences patterns, and supporting tools. Additionally, these results are used in other IT security related projects to provide solutions which could mitigate risk and ensure business continuity in an organization.

The CCMODE tool will be based on the D2R platform [43] and the broker technology. It allows uniform access to different specialized knowledge bases—one of them will support the IT security development process, others concern the IT product or system elaboration and their evaluation evidences, site-certification issues, selecting the right EAL for the TOE, and the internal organization and management of the entire development environment. The IT security development process is expressed by stereotyped UML classes in the Enterprise Architect tool [44], based on the data (enhanced generics—here discussed, components) retrieved from the knowledge base and dedicated plugin, and provided through the broker. The CCMODE different data are specified in RDF, RDFS, OWL and are identified with the use of URI (Uniform Resource Identifier). Ontologies as the upper layer have been elaborated using the Semantic works [37] tool—the whole system is implemented in the Java technology.

Summing up, the major contribution of the paper is to provide an ontology-based method and tool to elaborate and manage the specific security design patterns for the Common Criteria compliant IT security development. By applying a knowledge engineering approach to the Common Criteria domain, the author’s works aim at providing developers with: design patterns, methodology, tools and related knowledge, which all help to elaborate required security specifications.

The Common Criteria related design patterns were elaborated through the generalization of sensors functionality, architecture, known attacks, policies, *etc.* [3]. The set of design patterns for security specifications (enhanced generics) was validated on a few examples of sensors. The validations give input for the optimization of this set. Currently it encompasses representative items which are enough to specify security issues of intelligent sensors and sensors collaborating with each other in broader IT systems, though this set is open to new security problems, their solutions and technological advances. The introduced ontological representation of these patterns gives extra advantages brought by this approach. The security of sensors and sensors systems enables their new applications, where risk issues are important.
